# Sostdc1: A soluble BMP and Wnt antagonist that is induced by the interaction between myeloma cells and osteoblast lineage cells

**DOI:** 10.1016/j.bone.2019.02.012

**Published:** 2019-05

**Authors:** Z. Faraahi, M. Baud'huin, P.I. Croucher, C. Eaton, M.A. Lawson

**Affiliations:** a Institute for Cancer Sciences, University of Manchester, UK; b INSERM U957, EA 3822, Nantes, France; c Bone Biology Division, Garvan Institute of Medical Research, Sydney, Australia; d Department of Oncology and Metabolism, Medical School, University of Sheffield, UK

**Keywords:** CTNNB1, β-catenin, BMP, bone morphogenetic protein, B2M, β-microglobulin, chr, chromosome, Dkk1, Dikkopf-1, DAB, diaminobenzidine, Frz, frizzled, GAPDH, glyceraldehyde 3-phosphate dehydrogenase, GFP, green fluorescent protein, HGF, hepatocyte growth factor, HRP, horse radish peroxidase, IL, interleukin, LRP, lipoprotein-related protein, MM, multiple myeloma, OB, osteoblast, OGM, osteogenic medium, Runx, runt-related transcription factor, SMAD, C. elegans SMA/Drosophila mothers against decapentaplegic homolog, Sostdc1, sclerostin domain containing 1, SOST, sclerostin, sFRP, secreted frizzled-related protein, Wnt, wingless-related integration site, Sostdc1, Multiple myeloma, Bone morphogenic proteins, Wnt, Osteoblastogenesis

## Abstract

Multiple myeloma (MM) is characterised by destructive lytic bone disease, caused by induction of bone resorption and impaired bone formation. Our understanding of the molecular mechanisms responsible for osteoblast suppression, are limited. Using the 5T2MM murine model of MM we have previously shown that suppression of the activity of a known inhibitor of bone formation Dikkopf-1 (Dkk1) prevents the development of lytic bone disease. Here we have demonstrated that another potential inhibitor of bone formation, sclerostin domain containing 1 (Sostdc1) is expressed at low levels in MM and osteoblast lineage cells when these cells are grown separately in cell culture but its expression is significantly induced in both cell types when these cells are in contact. The distribution of Sostdc1 staining in bones infiltrated with 5TGM1 myeloma cells *in vivo* suggested its presence in both myeloma and osteoblast lineage populations when in close proximity. We have also shown that recombinant Sostdc1 inhibits both bone morphogenic proteins (BMP2 and 7) and Wnt signalling in primary osteoblasts and suppresses differentiation of these cells. Together, these findings suggest that Sostdc1 expression in 5TGM1-infiltrated bones as a result of the interaction between myeloma and osteoblast lineage populations, could result in suppression of osteoblast differentiation.

## Highlights

• Sostdc1 is a potent suppressor of osteoblast differentiation.
• Sostdc1in osteoblast and myeloma cells is induced when these cells interact.
• Sostdc1 down regulates Wnt-BMP signalling crosstalk in osteoblasts.
• Sostdc1 is present in myeloma-infiltrated bone disease and not present in normal bone.

## 1 Introduction

Recently, studies have shown that multiple myeloma (MM) cells arriving in bone occupy niches, where they interact with specific bone cell lineages [[1], [2], [3]]. Myeloma cells held within niches are in a mitotically dormant state and this is maintained until they are released, leading to the development of lytic bone disease in 90% of patients [2]. Dormancy allows single tumour cells establishing residency in bone time to adapt to new environmental influences and to avoid the effects of drugs that target proliferating cells. The composition of the niche and how this controls dormancy are not well understood, but there is evidence that osteoblast (OB) lineage cells are important components. Lytic bone disease associated with myeloma growth has, however, been extensively studied [4] and colonised bone is characterised by reduced osteoblastogenesis [5]. Osteolytic lesions arise in close proximity to the tumour, suggesting that close contact between myeloma cells and bone cells is required to influence bone remodelling [6]. A number of molecules have been implicated in the suppression of OB differentiation in MM including the wingless-related integration site (Wnt)-signalling inhibitor Dickkopf-related protein 1 (Dkk1) [7,8], secreted frizzled-related protein 2 (sFRP-2) [9,10], interleukin 7 (IL-7) [11,12] and hepatocyte growth factor (HGF) [11], IL-3 [13,14] and sclerostin (SOST) [15,16]. Antibodies to Dkk1 and SOST prevent the suppression of OB differentiation and the development of lytic bone disease *in vivo* [7,17]. However, there are likely to be other regulators that contribute to OB suppression in myeloma-induced bone disease [18].

In this study we aimed to identify other important factors involved in the development of myeloma-induced bone disease, using the 5TGM1 syngeneic murine model that develops osteolytic lesions in infiltrated bones [19]. SOST and Sostdc1 are proteins with ~55% homology in humans and both mediate suppression of bone morphogenic proteins (BMPs) and Wnt signalling [20]. In humans, the *SOST* gene is located on chromosome 17 (Sost: chr 12 in mouse) and *Sostdc1* on chromosome 7 (Sostdc1: chr 11 in mouse), the presence of the 2 genes being a result of past gene duplication where some division of product function has evolved [20]. The effective functional differences between the 2 proteins appears to result from the distribution of the expression of the genes with SOST being highly expressed in bone and Sostdc1 being expressed in the kidney, tooth buds, and lung tissue. Studies with knock-out mice show that loss of SOST expression results in sclerosteosis in the axial skeleton, while there is no general bone phenotype in Sostdc1 knock out mice apart from effects in the teeth including fusion and extra incisors [21]. The factors that control the expression of these genes in specific locations are not fully understood but it is suggested that BMPs/transforming growth factor βs and fibroblast growth factors, as well as vitamin D signalling, regulate transcription of both genes. The segregation of expression of each protein to different anatomical sites would suggest the need for control of action and that inappropriate expression in tissues could have deleterious effects, as suggested by recent studies of the formation of digits in experimental animals [22].

As Sostdc1 is a putative inhibitor of OB differentiation that is not expressed in adult bone, its presence in MM cells and in myeloma-infiltrated bones would make it an interesting candidate in the context of myeloma-induced bone disease. We have shown that MM and OB lineage cells produce little Sostdc1 until they are in close proximity to each other, when the protein is induced in both cell types. We subsequently evaluated the function of this protein in *in vitro* OB differentiation assays.

## 2 Materials and methods

### 2.1 Ethics statement

All procedures involving mice were conducted at the University of Sheffield, UK and were approved by the Home Office (PPL 40/3462) and the University of Sheffield's Animal Ethics Committee in accordance with the Animal [Scientific Procedures] Act 1986 and ARRIVE guidelines.

### 2.2 Calvarial primary OB isolation and differentiation

Mouse primary OB progenitor cells were isolated from the calvarial bones of 2 to 4 day old C57BLKaLwRij mice (Harlan, UK) using Collagenase I (1 mg/ml, Sigma Aldrich) digestion solution as previously described [23]. Isolated calvarial cultures were pooled and re-suspended in complete Minimum Essential Medium alpha (MEMα) (Invitrogen, UK), containing 10% foetal calf serum (FCS), 100 units/ml penicillin/100 μg/ml streptomycin. To differentiate OB progenitors, cells were seeded (6000 cells/cm^2^) for 72 h in complete MEMα and differentiated in osteogenic media (OGM): MEMα containing 4% FCS, 10 mM β-glycerol phosphate and 50 μg/ml ascorbic acid. In preliminary experiments, the basic growth/differentiation characteristics of the primary osteoblast progenitors was evaluated over time courses up to 15 days post-addition of OGM. These studies showed that the cultures could be maintained in 4% FCS and the presence of differentiation markers were first clearly observable on day 8 post treatment. This time point was used for subsequent studies evaluating the effects of Wnt3a, BMP2, BMP7 or BMPs with and without antagonists/inhibitors.

### 2.3 Murine 5TGM1 myeloma cells

Murine 5TGM1 wildtype and 5TGM1-GFP expressing myeloma cells (a kind gift from Dr. Oyajobi, University of Texas, San Antonio, USA) were maintained in complete RPMI medium as previously described [2].

### 2.4 Myeloma-OB co-cultures

OB progenitor cells were differentiated in culture plates or T175 flasks for 8 days. On day 8 of differentiation, 5TGM1-GFP cells were counted and co-cultured on the differentiating OB progenitors at a cell density of 12,000 cell/cm^2^ similar to the estimated OB progenitor cell number on day of 8 of differentiation. Cell seeding densities were previously determined following OB progenitor growth curves suggesting that OB cultures approximately doubled in DNA contents/cell number by day 8 of differentiation (data not shown). 5TGM1-GFP/OB progenitor cells were co-cultured for 24 h in complete RPMI media with the differentiating OB progenitors at the same cell density as in cultures of each cell line grown alone. This provided the opportunity for direct contact between OB progenitors and 5TGM1 cells.

### 2.5 Sostdc1 detection in 5TGM1 and OB cells by immunofluorescence

For immunofluorescent detection of Sostdc1 in cell cultures, cell adherence was facilitated using tissue culture grade poly-l-lysine polymer reagent to coat all plates. 5TGM1 wildtype cells were fixed with 4% formalin and permeabilised with 0.1% Triton X for 10 min treated with 10% normal goat serum for 30 min. Cells were stained with anti-Sostdc1 (1 μg/ml anti-Sostdc1 rabbit polyclonal antibody, Abcam) or an isotype control (1 μg/ml rabbit IgG polyclonal, Abcam), overnight at 4 °C. The next day, cells were stained with an anti-rabbit secondary antibody (6.6 μg/ml donkey anti-rabbit polyclonal IgG NorthernLights™ NL637-conjugated antibody, R&D Systems) for 1 h at room temperature. Cells were doubled stained with an anti-Syndecan-1 (CD138) (6.6 μg/ml anti-Syndecan-1 mouse monoclonal FITC-conjugated antibody, Abcam), a plasma cell marker, or an isotype control (6.6 μg/ml mouse IgG monoclonal FITC-conjugated antibody, eBiosciences) for 1 h. 4′,6-Diamidino-2-phenylindole, dilactate (DAPI) was used to visualise nuclear staining. Images of phase contrast, DAPI, CD138 and Sostdc1staining were visualised simultaneously and represented as a single stain on their own or merged as one image. Cellular staining was observed by AF6000LX software (Leica DM16000 inverted microscope).

### 2.6 Flow cytometry and cell sorting

Cells from populations grown alone were detached, pelleted, fixed with 4% formalin and permeabilised in 0.1% Triton-X for 10 min, before being treated with 10% normal donkey serum for 30 min to reduce non-specific binding of the secondary antibody. Cells were incubated with an anti-Sostdc1 antibody (1 μg/ml anti-Sostdc1 rabbit polyclonal antibody, Abcam) or isotype control antibody (1 μg/ml rabbit IgG polyclonal antibody, Abcam) for 30 min. Cell suspensions were incubated with a fluorescent-conjugated secondary antibody (2 μg/ml donkey anti-rabbit polyclonal IgG NorthernLights™ NL637-conjugated antibody, R&D Systems) for 30 min. Flow cytometric analysis was performed using the BD FACSCalibur™ platform and the data analysed using the Cell Quest software. 5TGM1-GFP cells were detected *via* FL1 fluorescence channel and OB progenitors through the FL4 fluorescence channel. Cell sorting of co-cultures was performed with the FACS Aria flow cytometer (BD Biosciences), in which 5TGM1-GFP and OB progenitor co-cultures were separated into OB progenitor and 5TGM1-GFP cell populations using GFP as a marker. In brief, 5TGM1-GFP and OB progenitor cells cultured alone were sorted first so that the correct gating could be applied for each individual population. Cells from OB-myeloma co-cultures were sorted into 1 ml of RPMI for approximately 30 min. 1 ml sorted cell populations were then split into two tubes containing 500 μl each; from which protein or RNA was extracted.

### 2.7 *In vivo* study to obtain naïve and 5TGM1-infiltrated bone tissue sections

All animals were housed in cages under standard conditions (12 h light/dark cycle) and were healthy and pathogen free at the start of the study. Once the study had commenced animals were monitored daily for any unexpected adverse effects. Animals were housed in groups and the numbers per group were determined using power calculations based on previous studies where reproducible statistical differences had been demonstrated (2).

Male 7–8 weeks old C57BL/KaLwRijHSD (C57BLKaLwRij) mice (Envigo, UK) were randomised based on weight (18–24 g) into 2 groups and injected intravenously *via* the tail vein with either 100 μl PBS (n = 4, naïve, non-tumour bearing control group) or 2 × 10^6^ 5TGM1-GFP expressing cells (n = 4, 5TGM1-bearing group). At the first signs of morbidity (after 3 weeks) all animals were anesthetized (100% w/v isoflurane & 2% oxygen by inhalation) for cardiac bleeding and sacrificed by cervical dislocation.

### 2.8 Detection of Sostdc1 in tissue sections by immunohistochemistry

Tibiae from naïve and 5TGM1-tumour bearing mice were fixed in 4% paraformaldehyde, decalcified, and paraffin embedded bone sections were cut. Antigen retrieval was done using 1:3 dilution of trypsin enzyme for 10 min. Sections were quenched with 10% H_2_O_2_ and blocked with 10% goat serum solution (Invitrogen) for 30 min before incubation with rabbit anti-Sostdc1 antibody (1 μg/ml anti-Sostdc1 rabbit polyclonal antibody, Abcam) over night at 4 °C followed by goat anti-rabbit biotinylated antibody (2 μg/ml goat anti-rabbit polyclonal biotinylated antibody, R&D Systems) for 30 min and incubation with streptavidin solution (3 μg/ml, ThermoFisher) for 30 min. Antibody-antigen specific staining was developed with DAB Chromogen kit (Vector Labs, UK). Tissue sections were counterstained with Gills haematoxylin and images captured using an Aperio® ScanScope slide scanner.

### 2.9 Western blot analysis

Protein was extracted from lysates of OB progenitor cells using a cell mammalian lysis kit (Sigma, UK) and 10 μg loaded onto an SDS-PAGE gels. Electrophoresed proteins were transferred onto a polyvinylidene fluoride membrane (Milipore, Bedford, USA) and nonspecific binding sites blocked for 1 h with 3% bovine serum albumin (BSA). Blots were probed overnight (4 °C) with polyclonal antibodies for Sostdc1 (3 μg/ml anti-Sostdc1 rabbit polyclonal antibody, Abcam), phosphorylated Smads 1, 5 and 8 proteins (3 μg/ml, Phospho-Smad1/Smad5/Smad8 rabbit polyclonal antibody, Cell Signalling), β-catenin (5 μg/ml, anti-β-catenin rabbit polyclonal antibody, Abcam) and GAPDH (2 μg/ml, anti-GAPDH mouse monoclonal antibody, Abcam). Membranes were incubated with a HRP-conjugated secondary antibody specific to rabbit (1 μg/ml, goat anti-rabbit IgG HRP-conjugated antibody, Life Technologies Novex® or mouse (1 μg/ml, goat anti-mouse IgG HRP-conjugated antibody, Santa Cruz) for 1 h. A chemiluminescent substrate (Super Signal West Dura, Thermo Fisher) was used for detection of specific proteins on X-ray films (Kodak). Protein bands on X-ray films were analysed using the GS-710 Calibrated Imaging Densitometer (Biorad) and the Quantity One software. An intensity threshold of 1 was set on all lanes on the same blot and any background noise minimised to obtain the relative intensity (RI) measure for individual bands. Measurements of RI for target proteins were normalised to corresponding GAPDH RI levels.

### 2.10 OB progenitor mineralisation

The mineralisation of OB progenitors treated with Sostdc1 in the presence or absence of Wnt3a, BMP2 or BMP7 was assessed using Alizarin red staining. This is a definitive functional marker of full osteoblast differentiation. OB cultures were fixed in 100% ethanol for 1 h and stained with 1% Alizarin red stain for 20 min. Culture plates were scanned (V800 Scanner, Epson UK) and the percentage area of mineralisation per well was quantified using ImageJ (http://imagej.nih.gov/ij/; National Institutes of Health, Bethesda, MD, USA) as described previously [24].

### 2.11 5TGM1 and OB progenitor cells RNA isolation and RT-PCR

RNA was isolated from 5TGM1 and OB progenitor cells using the ReliaPrep RNA cell Miniprep kit (Promega). Complementary DNA (cDNA) was synthesized using 0.5–1 μg of total RNA and gene expression quantified using TaqMan® assays for quantitative RT-PCR (qRT-PCR) analysis by SDS2.2.1. (Applied Biosystems): *Runx2* (Mm00501584_m1), β-catenin (*CTNNB1)* (Mm00483039_ml) and β2 microglobin (*B2M)* (Mm00437762_m1). Absolute and relative gene expression quantification was normalised to the house keeping *B2M* gene using the formula ΔCT = CT_target_ − CT_housekeeping_. Presence of specific PCR products was verified using 1.5% agarose-TBE gel electrophoresis. Gels were loaded with 10 μl of DNA ladder (full range 100 BP Norgen) and 10 μl of PCR product and electrophoresis performed at 100 V for 30 min. DNA fragments were inspected under UV light using the Gel Doc XR+ System and the Quantity One software (Bio-Rad). PCR products obtained from OB progenitor and 5TGM1 cultures and co-cultures were sequenced using the Applied Biosystems' 3730 DNA to verify Sostdc1 product identity (Sostdc1: forward 5-CCGTCATGCTTCTCAGTTTC and Sostdc1: reverse 3-GCTGTCACACTCCAAGGGCC). Sequencing results were analysed using FinchTv software version 1.4.0 and the base pair sequences were assessed for nucleotide similarities to Sostdc1.

### 2.12 Interferometry kinetics for Sostdc1 interaction binding partners

The Bio-Layer Interferometry (BLI) (ForteBio) (www.ForteBio.com) using Amine reactive 2nd generation (AR2G) biosensors were used to evaluate interactions between recombinant Sostdc1 and low density lipoprotein receptor-related protein 6 (LRP6), BMP2 or BMP7. Interferometry data were globally fit to a simple 1:1 Langmuir model where one ligand molecule interacts with one analyte molecule and the affinities and rate constants calculated (Octet software, Version 6.4, ForteBio). To produce a complete kinetic prolife for Sostdc1 and its associated binding partner, the interaction was measured at multiple analyte concentrations ranging from 0 to 100 μg/ml and the data used for Langmuir model fitting. The association and dissociation responses were baseline corrected and processed using the Octet Software (Version 6.4, ForteBio). The individual signal responses at each concentration were calculated and the measured affinity of the interaction *K*D (M) between the two proteins reported by the BLItz Pro™ software.

### 2.13 Statistical analysis

All data are presented as mean ± SEM of at least three experiments. Statistical analysis was performed using a Students unpaired *t-*test (Mann-Whitney if not parametric) and one-way ANOVA, using Prism 6 software. *P < 0.05 was considered statistically significant.

## 3 Results

### 3.1 Sostdc1 levels are increased in myeloma and OB progenitor co-cultures in both cell types

Our initial hypothesis was that Sostdc1 was produced by myeloma cells and not by OB lineage cells. This was tested using the syngeneic 5TGM1 murine model of MM, which results in myeloma-induced bone disease in mice, and *in vitro* in cultures of murine 5TGM1 myeloma cells and primary murine OB progenitor cells. Each cell type was grown alone and in co-culture.

**Fig. 1:**
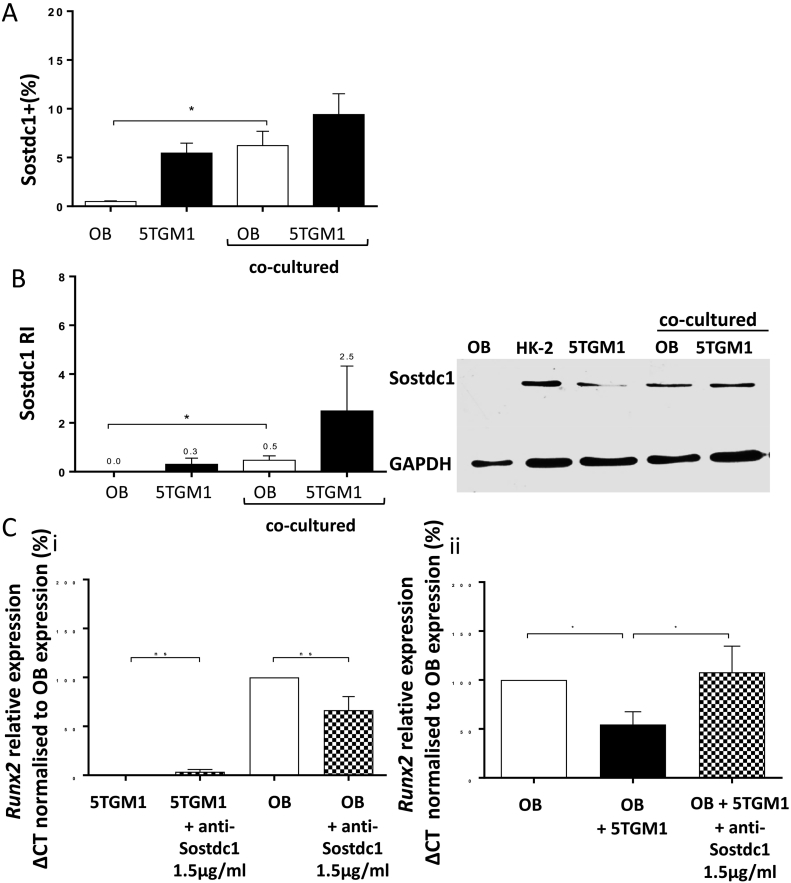
Sostdc1 protein levels are increased by myeloma cells-OB progenitor interaction and blocking Sostdc1 reverses myeloma-induced suppression of OB differentiation: (A) Flow cytometry analysis of 5TGM1-GFP cells and OB progenitors (alone or in co-culture) for the expression of murine Sostdc1 protein. (B) Western blot analysis of cell lysates from 5TGM1-GFP cells and OB progenitors (alone or in co-culture) illustrating the level of Sostdc1 protein. The relative intensity (RI) of the Sostdc1 protein band was normalised to the RI of corresponding GAPDH protein band in the same sample. HK2 cells were used as a positive control for Sostdc1 expression. (C) Quantitative RT-PCR analysis of *Runx2* gene expression in 5TGM1-GFP cells and OB progenitors (alone or in co-culture), in the absence or presence of anti-Sostdc1 antibody. *N* = 3 independent experiments, Student *t*-test, One-way ANOVA, *P < 0.05.

Flow cytometric analysis of cell cultures showed that ~5% of the 5TGM1 cells and <1% of the OB progenitors grown alone were Sostdc1 positive but this increased significantly to ~6% in the OB progenitors isolated from co-cultures (*P* = 0.043, Fig. 1A). To test whether Sostdc1 protein levels were increased in co-cultured cell populations, cells were either grown alone or sorted from co-cultures into OB progenitor or 5TGM1 myeloma cell populations and Sostdc1 protein levels were evaluated by Western blotting (Fig. 1B). These studies confirmed the presence of Sostdc1 in the co-cultured OB progenitor population in comparison to OB progenitors cultured alone (*P* = 0.028), where Sostdc1 protein was near the limit of detection. There was also an increase in Sostdc1 protein in the 5TGM1 cells sorted from the co-cultures compared to 5TGM1 cells grown alone, although this was not statistically significant.

To test whether OB progenitor differentiation was suppressed by 5TGM1 derived Sostdc1, the levels of expression of the OB marker *Runx2* was evaluated in populations grown alone and in co-culture ±1.5 μg/ml anti-Sostdc1 neutralising antibody. Data showed that 5TGM1 cells cultured alone did not produce detectable levels of *Runx2* (Fig. 1C). *Runx2* expression was detected in differentiating OB progenitors cultured alone and expression was reduced in these cells in the presence of 5TGM1 cells (*P* = 0.023). Treatment of 5TGM1-OB progenitor co-cultures with anti-Sostdc1 antibody reversed this effect, restoring *Runx2* levels in OB progenitors (*P* = 0.028).

**Fig. 2:**
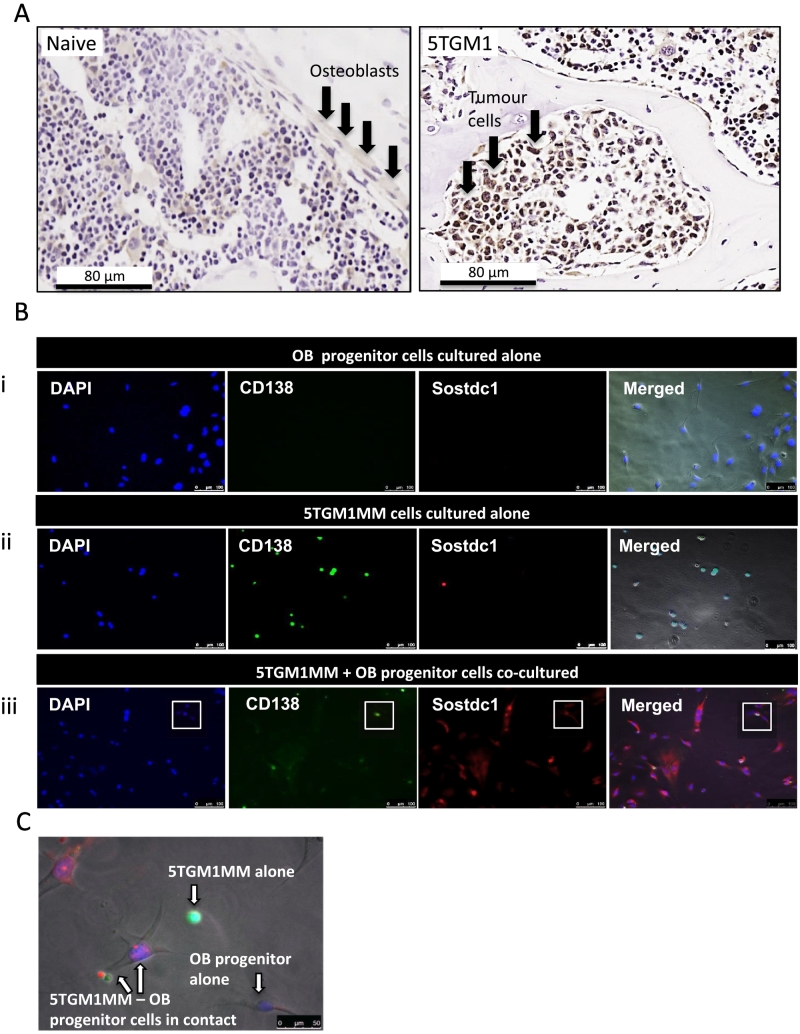
Sostdc1 was upregulated in 5TGM1 tibiae sections and 5TGM1-OB progenitor co-cultures: Immunoassays and end-point PCR were performed to detect Sostdc1 in 5TGM1-GFP cells and differentiating OB progenitors (alone and in co-culture). (A) Immunohistochemistry for Sostdc1 in naïve and 5TGM1-infiltrated tibia sections. Arrows indicate osteoblasts (naïve, left image) or tumour cells (5TGM1, right image). (B) Confocal microscopy for Sostdc1 in OB progenitors cultured alone (i) 5TGM1-GFP (CD138 positive cells) cultured alone (ii) or in co-culture (iii). 5TGM1/OB progenitors in contact highlighted in white squares. (C) High power field of co-cultures stained with DAPI and with antibodies for CD138/Sostdc1 merged. Isolated and associated cells are highlighted. All figures are representative of 3 independent experiments.

Immunohistochemistry was used to compare the presence of Sostdc1 protein expression in naïve (non-tumour) murine tibiae sections to those infiltrated with 5TGM1 cells. Sostdc1 protein was present in 5TGM1-infiltrated tibiae sections and absent in sections obtained from naive animals (Fig. 2A). Sostdc1 staining was strongest within the 5TGM1 cells themselves in these sections, although it was difficult to identify any OB cells in close proximity to 5TGM1 colonies at the time points studied.

Immunofluorescent microscopy was used to determine the presence of Sostdc1 protein in OB progenitor cells and 5TGM1 cells cultured alone and in co-culture. OB progenitors stained with anti-Sostdc1 were negative, whilst 5TGM1 cells were weakly positive for staining with this antibody (Fig. 2B). Co-culture of 5TGM1 cells with OB progenitor cells resulted in clear Sostdc1 staining in both cell types. Sostdc1 staining in these co-cultures appeared to be both intracellular and membrane bound (Fig. 2B & C). Staining for Sostdc1 was distinct and intense where there was direct contact between the 5TGM1 cells and OB progenitor cells. In all cultures, there was no staining with the isotype control antibodies (results not shown).

### 3.2 Recombinant Sostdc1 suppressed Wnt and BMP-induced differentiation and mineralisation in OB progenitors

**Fig. 3:**
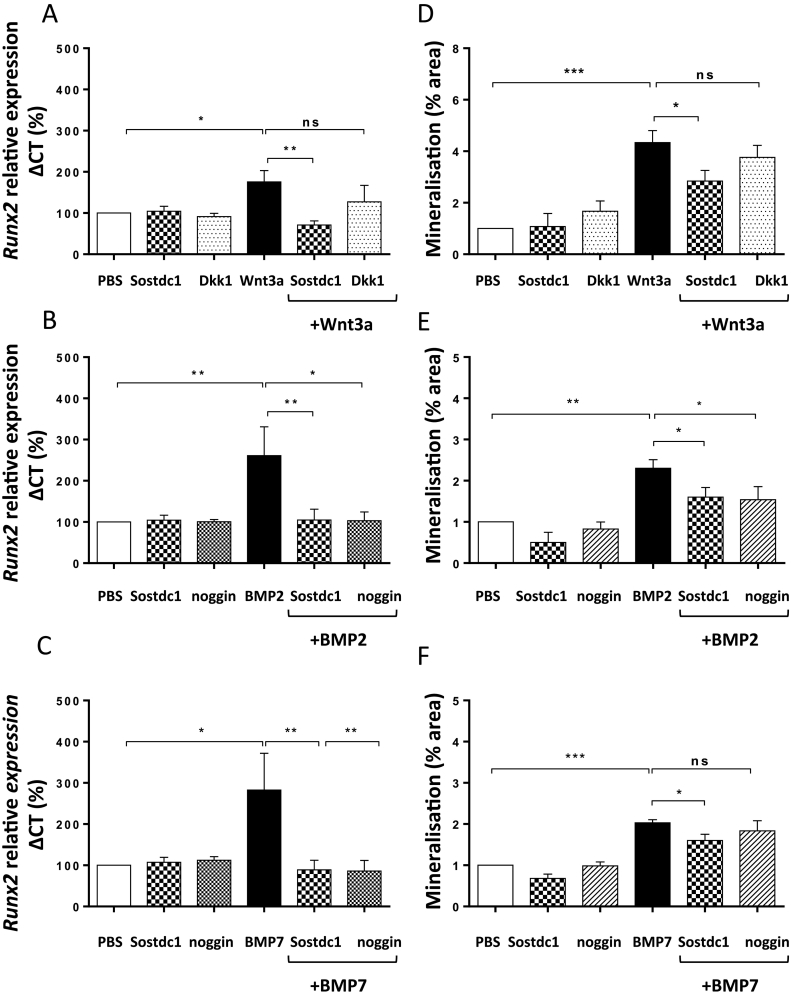
Sostdc1 reduces OB progenitor differentiation induced by Wnt and BMP ligands: Differentiating OB progenitors were treated with Wnt3a, BMP2 or BMP7. Dkk1 and noggin were used as known Wnt and BMP antagonist controls, respectively. Quantitative RT-PCR of *Runx2* mRNA relative expression to B2M in differentiating OB progenitors treated with Wnt3a (A), BMP2 (B) and BMP7 (C) alone or in the absence or presence of Sostdc1, Dkk1 or noggin. Mineralisation of OB progenitors treated with Wnt3a (D), BMP2 (E) and BMP7 (F) in the absence or presence of Sostdc1, Dkk1 or Noggin. Results are expressed as the % area of the well that was mineralised. *N* = 4 independent experiments. One-way ANOVA, *P < 0.05, **P < 0.01, ***P < 0.001.

Differentiating OB lineage cells isolated from the calvaria of neonatal mice and maintained in cell culture in low levels of FCS were challenged with murine recombinant Sostdc1. Quantitative RT-PCR was used to assess the effect of Sostdc1 on Wnt and BMP-induced *Runx2* gene expression (Fig. 3). The effect of Dkk1 on Wnt3a-induced *Runx2* gene expression and noggin on BMP-induced expression were also assessed, as they are known Wnt and BMP antagonists respectively. The optimal concentration of the recombinant proteins used as stimulants or antagonists of the Wnt/BMP pathway was determined *via* dose response viability assays (data not shown). The IC50 and Hill Slope of the dose-response curve were used to calculate the concentration at which 50% of OB progenitor cells survived following treatment with a stimulant or antagonist. These studies showed that on their own, Sostdc1, noggin and Dkk1 had no effect on *Runx2* gene expression (Fig. 3). Wnt3a on its own induced approximately two-fold increase in *Runx2* gene expression (*P* = 0.035), and Sostdc1 (250 ng/ml) inhibited this Wnt3a-induced *Runx2* gene expression (*P* = 0.005, Fig. 3A). Dkk1 had no significant effect on Wnt-induced *Runx2* gene expression during OB progenitor differentiation. BMP2 induced *Runx2* gene expression of OB progenitor differentiation and Sostdc1 inhibited this inductive effect (*P* = 0.005, Fig. 3B). Noggin had a similar effect to Sostdc1, down regulating BMP2-induced *Runx2* gene expression levels in differentiating OB progenitors (*P* = 0.049, Fig. 3B). BMP7 induced *Runx2* expression in the early stages of OB progenitor differentiation (*P* = 0.013) and Sostdc1 was able to significantly reverse this effect (*P* = 0.008, Fig. 3C). In the presence of BMP7, noggin had a similar suppressive effect to Sostdc1 on *Runx2* gene expression in the early stages of OB progenitor differentiation (*P* = 0.008, Fig. 3C).

Alizarin Red staining was used to investigate the effect of Sostdc1 on OB progenitor mineralisation in the presence of Wnt3a, BMP2 or BMP7. Wnt3a (50 ng/ml) significantly stimulated mineralisation of OB progenitor cells (*P* < 0.0001) and addition of Sostdc1 to Wnt3a treated OB progenitors resulted in a significant reduction of OB progenitor mineralisation (*P* = 0.016, Fig. 3D). Dkk1 had no suppressive effects on Wnt3a-induced mineralisation under the same experimental conditions. Analysis of Alizarin Red staining showed that both BMP2 and BMP7 induced OB differentiation (*P* = 0.002, Fig. 3E, *P* = 0.001 Fig. 3F, respectively) compared to controls. The BMP2 and BMP7-induced calcium mineralisation was inhibited in the presence of Sostdc1 (*P* = 0.044, Fig. 3E**,** *P* = 0.019 Fig. 3F). The addition of noggin in the presence of BMP had no significant suppressive effects on BMP2- or BMP7-induced mineralisation under the same experimental conditions.

### 3.3 Sostdc1 inhibited acute Wnt and BMP-induced intracellular signalling in OB progenitors

To determine the effect of Sostdc1 on Wnt and BMP-induced intracellular signalling, the status of downstream signalling molecules in these pathways were separately assessed following addition of recombinant Sostdc1 treatment to OB cultures. OB progenitor cells were differentiated and stimulated with single or combination protein treatments for 20 min, after which OB progenitor cultures were lysed. To assess the effect of Sostdc1 on Wnt-induced intracellular signalling, β-catenin protein levels were determined using Western blot analysis. The effect of Sostdc1 on BMP2- and BMP7-induced downstream signalling was further analysed *via* quantification of phosphorylated levels of Smads 1, 5 and 8 proteins. Dkk1 (100 ng/ml) and noggin (100 ng/ml) were used as known inhibitors of BMP signalling.

**Fig. 4:**
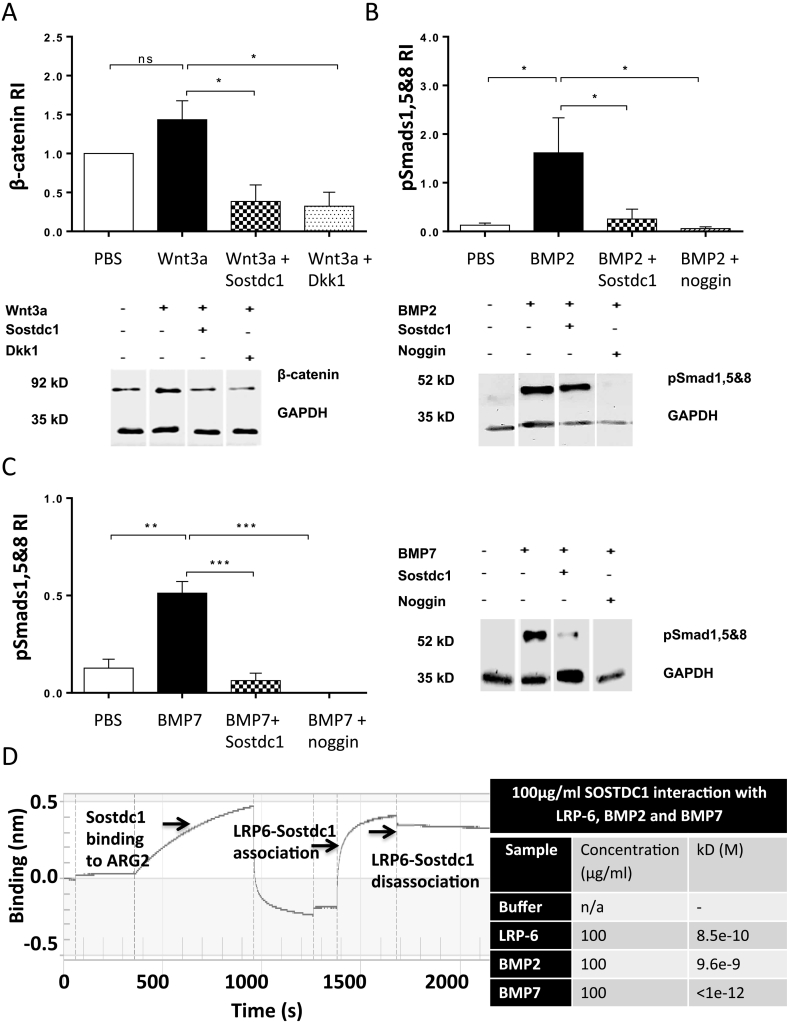
Sostdc1 reduces Wnt/BMP signalling and binds to LRP6, BMP2 and BMP7 proteins: Differentiating OB progenitors were treated with Wnt3a, BMP2 or BMP7 in the absence or presence of recombinant murine Sostdc1 protein for 20 min. Western blot analysis and quantification of cell lysates from OB progenitors stimulated by Wnt3a (A), BMP2 (B) or BMP7 (C) in the absence or presence of Sostdc1, Dkk1 or noggin illustrating the level of β-catenin (A) or Phospho-Smads 1, 5 & 8 (B and C). Kinetic analysis and binding affinity of Sostdc1 binding to LRP6, BMP2 and BMP7 using the Blitz Analysis system. (D) 100 μg/ml of Sostdc1 bound to rmLRP6, BMP2 and BMP7, and had the highest binding affinity for BMP7. N = 4 independent experiments. One-way ANOVA, *P < 0.05, **P < 0.01. ***P < 0.001.

Western blot analysis showed that in the presence of Wnt3a, Sostdc1 suppressed β-catenin protein levels in OB progenitor cultures (*P* = 0.019, Fig. 4A). Dkk1 protein had a similar suppressive effect on Wnt-induced β-catenin protein levels (*P* = 0.012). Densitometry data showed that BMP2 and BMP7 both induced Smads 1, 5 and 8 protein levels and Sostdc1 reversed this effect (BMP2 *P* = 0.045, Fig. 4B; BMP7 *P* = 0.0002, Fig. 4C). Similarly, noggin also down-regulated BMP-induced Smads 1, 5 and 8 protein levels in the early stages of OB progenitor differentiation (BMP2 *P* = 0.037, Fig. 4B; BMP7 *P* < 0.0001, Fig. 4C).

The kinetics of Sostdc1 with potential binding partners was assessed using interferometry. The binding affinity (*K*D) of Sostdc1 for the Wnt receptor recombinant murine LRP6 was 8.502 · 10^−10^ M *K*D, Sostdc1 for BMP2 was 9.569 · 10^−9^ M *K*D and Sostdc1 for BMP7 was calculated at <1.0 · 10^−12^ M *K*D (Fig. 4D). The affinity of Sostdc1 for the LPR6 receptor protein was one-fold higher compared to the affinity of Sostdc1 for BMP2 ligand protein. This data show that Sostdc1 had the highest binding affinity for the ligand BMP7 protein out of all three Sostdc1-protein interactions. The Sostdc1 protein had a three-fold higher binding affinity for BMP7 compared to LRP6.

### 3.4 Sostdc1 down regulated Wnt-BMP signalling crosstalk in OB progenitors

**Fig. 5:**
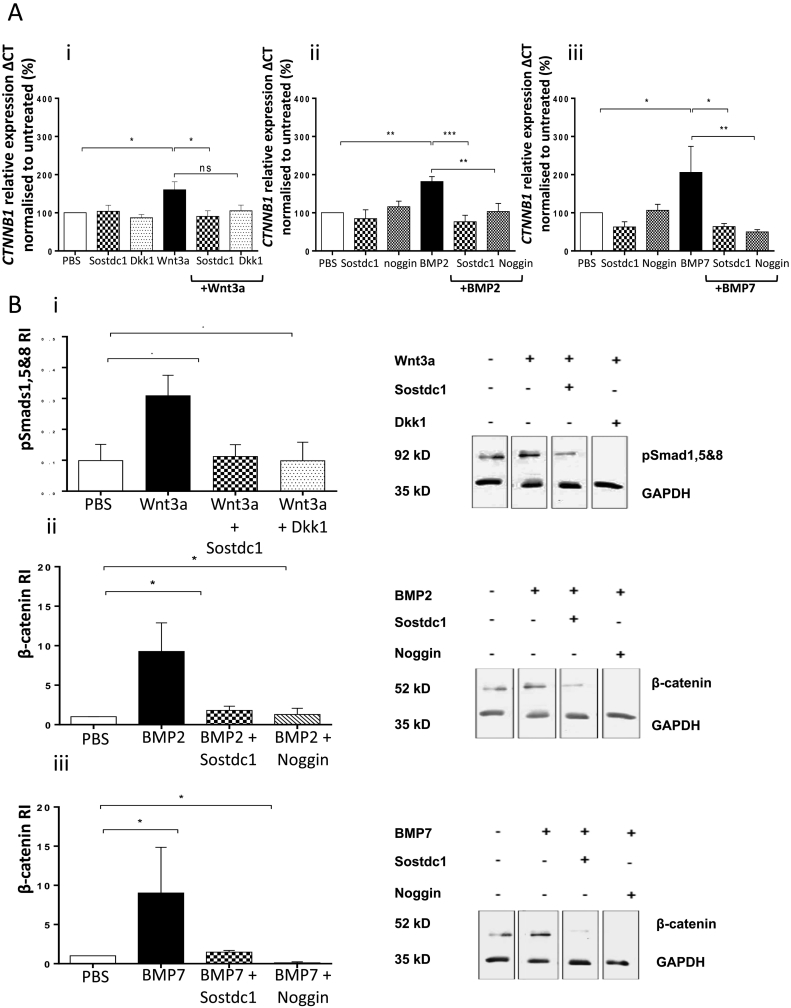
Sostdc1 inhibits BMP-induced β-catenin (*CTNNB1)* expression in differentiating OB progenitors: (A) Quantitative RT-PCR of *CTNNB1* in OB progenitors cultured with Wnt3a, BMP2 or BMP7 in the presence or absence of Sostdc1, Dkk1 or Noggin. Data represent the relative expression of *CTNNB1* to *B2M*. (B) Western blot analysis of cell lysates from OB progenitors stimulated by Wnt3a (i), BMP2 (ii) or BMP7 (iii) in the absence or presence of Sostdc1, Dkk1 or noggin illustrating the level of Phospho-Smads 1, 5 & 8 (i) or β-catenin (ii and iii). N = 4 independent experiments, One-way ANOVA, *P < 0.05, **P < 0.01 and ***P < 0.001.

In studies to determine potential crosstalk between BMP and Wnt signalling pathways, the effects of Wnt3a alone on β-catenin (*CTNNB1*) expression was initially assessed. The levels of *CTNNB1* and *B2M* expression were quantified by qRT-PCR in OB progenitor cells. Wnt3a (50 ng/ml) significantly increased *CTNNB1* expression in differentiating OB progenitors (*P* = 0.034, Fig. 5Ai). In the same experiments, Sostdc1 (250 ng/ml), but not Dkk1 significantly reduced Wnt3a-induced *CTNNB1* levels (*P* = 0.0142). The *CTNNB1* expression by OB progenitors was not affected in the presence of Sostdc1 or Dkk1 alone. Further analysis of *CTNNB1* expression in BMP-stimulated OB progenitors showed that BMP2 (30 ng/ml) and BMP7 (30 ng/ml) induced *CTNNB1* gene expression of OB progenitor differentiation on a similar level to that observed in cultures treated with Wnt3a (*P* = 0.006 and *P* = 0.025). In the presence of BMP2, Sostdc1 reduced *CTNNB1* levels in OB progenitors (*P* < 0.0001, Fig. 5Aii). Noggin (100 ng/ml) also suppressed BMP2-induced *CTNNB1* levels in OB progenitors (*P* = 0.006). BMP7-induced *CTNNB1* expression was also reduced in the presence of Sostdc1 (*P* = 0.010) and noggin reduced BMP7-induced *CTNNB1* transcript levels (*P* = 0.008, Fig. 5Aiii).

Western blot analysis was performed to determine whether Wnt3a increased phosphorylated Smads 1, 5 and 8 protein levels downstream of the BMP signalling pathway in differentiating OB progenitors. Wnt3a (50 ng/ml) acutely induced Smads 1, 5 and 8 protein phosphorylation in differentiating OB progenitors (*P* = 0.032, Fig. 5B). In the same experiments, Sostdc1 suppressed Wnt3a-induced phosphorylated Smads 1, 5 and 8 protein levels (*P* < 0.029) to a similar extent as Dkk1 (*P* = 0.037).

The level of β-catenin in differentiating OB progenitors was assessed following stimulation with BMP2 or BMP7 proteins. Both BMP2 and BMP7 were able to significantly induce β-catenin protein levels (*P* < 0.05). Sostdc1 and noggin both significantly suppressed BMP2 and BMP7-induced β-catenin in OB progenitor differentiation (*P* < 0.05) and β-catenin protein levels were not affected in the presence of Sostdc1 or noggin alone.

## 4 Discussion

Our studies, using a murine model of MM, show that Sostdc1 is present in the bone marrow of tibiae infiltrated with myeloma cells but not in the bone marrow of non-tumour bearing (naïve). The antibody used in these analyses and in studies with cell cultures to detect Sostdc1 was specific for this protein and was raised to a unique epitope not present on sclerostin. The presence of Sosdc1 in myeloma-infiltrated bones is novel and unexpected finding as this protein is not normally expressed in bone [20]. While immunohistochemistry showed protein expression within the 5TGM1 myeloma cells, it should be noted that the bones examined were well infiltrated at the time of sacrifice, a point where there were few visible osteoblastic cells remaining near to the myeloma colonies. This is not surprising since our studies have shown that recombinant Sostdc1 has potent effects in suppressing OB differentiation, specifically interfering with known pathways that drive this process. We initially thought that the myeloma cells themselves were the sole source of this protein in lesions. However, our *in vitro* studies showed that myeloma cells produced very low levels of Sostdc1 and the protein is undetectable in OB progenitor cells, when these cells are grown alone. In co-cultures, Sostdc1 expression and protein levels were induced in both cell types. We showed that in the co-culture studies, a marker of OB differentiation *Runx2* gene expression, was inhibited by the presence of 5TGM1 myeloma cells and that this effect could be reversed by co-treatment of cultures with a blocking antibody for Sostdc1, indicating that the levels of Sostdc1 generated in co-cultures are biologically active. Together these findings support the concept that Sostdc1 produced by interacting 5TGM1 and OB lineage cells *in vivo*, would induce the loss of OBs from bone. This alteration in the bone microenvironment may have effects on MM interactions with bone populations affecting myeloma cell survival and dormancy [1]. Interestingly, a number of studies have already shown that BMPs induce apoptosis and suppress proliferation of human myeloma cells [[25], [26], [27]]. Our studies would imply that Sostdc1 present in myeloma-infiltrated bones could suppress the above reported effects on apoptosis in MM by competitive binding to BMPs.

In *in vitro* studies we tested the hypothesis that Sostdc1 antagonises Wnt and BMP-induced differentiation and acute signalling in OB progenitors. Our data supported this hypothesis and showed that Sostdc1 reduced Wnt3a, BMP2 and BMP7-induced *Runx2* gene expression and calcium mineralisation of OB progenitors. These findings are similar to those of Laurikkala et al., which found the mouse orthologue of Sostdc1 inhibited both Wnt and BMP-induced MC3T3-E1 differentiation [28]. Interestingly though in our studies, Dkk1 had no significant effect on Wnt3a stimulated *Runx2* expression. However, since Sostdc1 acts on osteoblast progenitors and Dkk1 is secreted from mature osteoblasts, to inhibit Wnt signalling of osteoblast precursors [29], it is entirely possible that the time points that we see significant suppressive effects of Sostdc1 differ from those of Dkk1.

We also showed that in the presence of Wnt3a, Sostdc1 reduced β-catenin levels in OB progenitor cells. The suppression of Wnt3A induced a reduction in total β-catenin protein levels as well as decreases in the levels of activated (phosphorylated) forms of this protein in cultures of human osteoblastic cells in the presence of Sostdc1 (Supplementary Fig. 1). Similarly, the addition of Sostdc1 in the presence of BMP ligands reversed BMP-induced Smad phosphorylation. These observations suggest that the activities of Sostdc1 modulate both signalling systems involved in OB differentiation. Other studies have demonstrated a synergistic relationship between BMP and β-catenin during OB differentiation [[30], [31], [32]]. Secreted molecules such as cerberus and sclerostin, also inhibit OB activity by binding BMP/Wnt ligands [33] and receptors [34]. However, the regulatory role of Sostdc1 on Wnt-BMP crosstalk in differentiating OB progenitors has not been studied. Our data showed that treatment of differentiating OB with Wnt3a increased Smad phosphorylation and treatment with BMP2/7 enhanced Wnt signalling *via* β-catenin modulation. Similarly Zhang et al. reported that Wnt3a increased transcriptional activity of a BMP/Smad reporter and that co-treatment with a known inhibitor of BMP signalling, noggin, inhibited this effect [34]. Here we have shown that Sostdc1 negatively affected Wnt-BMP cooperation mediated by β-catenin-and regulatory-Smads in OB progenitors.

Lintern and Guidato et al. showed Sostdc1 may have separate domains for Wnt and BMP interaction, showing the independent binding capabilities of Sostdc1 to LRP6 receptor and BMP ligands [35]. Our interferometry data showed recombinant Sostdc1 had a highest binding affinity for BMP7, followed by LRP6 and lastly BMP2. These findings provide direct evidence that Sostdc1 has the capacity to interact with both BMP and Wnt signalling systems but may suggest selectivity for individual components although it can act as an effective antagonist for both pathways.

Our studies suggest Sostdc1 antagonises Wnt-BMP signalling in the early phase of OB differentiation. *In vitro* studies have suggested that Dkk1 mainly affects the function of mature OB cells and drives pluripotent cells to differentiate to OB lineages [36]. Rawadi et al. demonstrated that blocking Wnt/LRP5 signalling with Dkk1 in mesenchymal stem cells inhibited BMP2-induced alkaline phosphatase activity [32]. Dkk1 has also been shown to target BMPRIA, a BMP receptor in OBs, and mediate suppression of BMP signalling in mature bone. As Sostdc1 and Dkk1 both target the LRP/Frz-Wnt receptors and regulate Wnt-BMP crosstalk in OB progenitors, either molecule may be active at various stages in the development of myeloma-induced lytic bone disease: Dkk1 having a role in targeting mature OB cells, while Sostdc1 affects the maturation of OB progenitors.

In conclusion, we have shown that Sostdc1 is a potent suppressor of OB differentiation *in vitro* and that the production of this protein in OBs and myeloma cells is induced when these cells interact with each other. This is the first report showing the induced production of this protein by MM-OB interactions. These findings may suggest that targeting the induced production of Sostdc1 in MM infiltrated bones could have benefits in suppressing disease progression in MM.

The following is the supplementary data related to this article.

Supplementary Fig. 1 Sostdc1 suppressed Wnt3a-induced active and total β-catenin levels: Saos2 cells were treated with 50 ng/ml Wnt3a in the presence or absence of 250 ng/ml Sostdc1 or 100 ng/ml Dkk1 recombinant protein. Total and phosphorylated (p) β-catenin protein levels were assessed by western blotting (A). Multiple comparison analysis was used to assess the effect of Sostdc1 or Dkk1 on Wnt3a-induced active β-catenin (B) and pβ-catenin protein levels (C). Western blot image is representative of three independent experiments. One-way ANOVA, *P < 0.05, **P < 0.01 and ***P < 0.001. Supplementary Fig. 1

Supplementary material Image 1

## References

[1] Collette N.M., Yee C.S., Murugesh D., Sebastian A., Taher L., Gale N.W., Economides A.N., Harland R.M., Loots G.G. (2013). Sost and its paralog Sostdc1 coordinate digit number in a Gli3-dependent manner. Dev. Biol..

[2] Lawson M.A., McDonald M.M., Kovacic N., Hua Khoo W., Terry R.L., Down J., Kaplan W., Paton-Hough J., Fellows C., Pettitt J.A., Neil Dear T., Van Valckenborgh E., Baldock P.A., Rogers M.J., Eaton C.L., Vanderkerken K., Pettit A.R., Quinn J.M., Zannettino A.C., Phan T.G., Croucher P.I. (2015). Osteoclasts control reactivation of dormant myeloma cells by remodelling the endosteal niche. Nat. Commun..

[3] Croucher P.I., McDonald M.M., Martin T.J. (2016). Bone metastasis: the importance of the neighbourhood. Nat. Rev. Cancer.

[4] Munne P.M., Tummers M., Jarvinen E., Thesleff I., Jernvall J. (2009). Tinkering with the inductive mesenchyme: Sostdc1 uncovers the role of dental mesenchyme in limiting tooth induction. Development.

[5] Collette N.M., Genetos D.C., Murugesh D., Harland R.M., Loots G.G. (2010). Genetic evidence that SOST inhibits WNT signaling in the limb. Dev. Biol..

[6] Toscani D., Bolzoni M., Accardi F., Aversa F., Giuliani N. (2015). The osteoblastic niche in the context of multiple myeloma. Ann. N. Y. Acad. Sci..

[7] Tian E., Zhan F., Walker R., Rasmussen E., Ma Y., Barlogie B., Shaughnessy J.D. (2003). The role of the Wnt-signaling antagonist DKK1 in the development of osteolytic lesions in multiple myeloma. N. Engl. J. Med..

[8] Rachner T.D., Gobel A., Benad-Mehner P., Hofbauer L.C., Rauner M. (2014). Dickkopf-1 as a mediator and novel target in malignant bone disease. Cancer Lett..

[9] Oshima T., Abe M., Asano J., Hara T., Kitazoe K., Sekimoto E., Tanaka Y., Shibata H., Hashimoto T., Ozaki S., Kido S., Inoue D., Matsumoto T. (2005). Myeloma cells suppress bone formation by secreting a soluble Wnt inhibitor, sFRP-2. Blood.

[10] Habibi H., Abroun S., Hajifathali A., Soleimani M., Kaviani S., Kalantari N., Eslahchi S. (2013). Osteogenic inhibition in multiple myeloma. Cell J..

[11] Standal T., Abildgaard N., Fagerli U.M., Stordal B., Hjertner O., Borset M., Sundan A. (2007). HGF inhibits BMP-induced osteoblastogenesis: possible implications for the bone disease of multiple myeloma. Blood.

[12] Nierste B.A., Glackin C.A., Kirshner J. (2014). Dkk-1 and IL-7 in plasma of patients with multiple myeloma prevent differentiation of mesenchymal stem cells into osteoblasts. Am. J. Blood Res..

[13] Lee J.W., Chung H.Y., Ehrlich L.A., Jelinek D.F., Callander N.S., Roodman G.D., Choi S.J. (2004). IL-3 expression by myeloma cells increases both osteoclast formation and growth of myeloma cells. Blood.

[14] Ehrlich L.A., Chung H.Y., Ghobrial I., Choi S.J., Morandi F., Colla S., Rizzoli V., Roodman G.D., Giuliani N. (2005). IL-3 is a potential inhibitor of osteoblast differentiation in multiple myeloma. Blood.

[15] McDonald M.M., Reagan M.R., Youlten S.E., Mohanty S.T., Seckinger A., Terry R.L., Pettitt J.A., Simic M.K., Cheng T.L., Morse A., Le L.M.T., Abi-Hanna D., Kramer I., Falank C., Fairfield H., Ghobrial I.M., Baldock P.A., Little D.G., Kneissel M., Vanderkerken K., Bassett J.H.D., Williams G.R., Oyajobi B.O., Hose D., Phan T.G., Croucher P.I. (2017). Inhibiting the osteocyte-specific protein sclerostin increases bone mass and fracture resistance in multiple myeloma. Blood.

[16] Delgado-Calle J., Anderson J., Cregor M.D., Condon K.W., Kuhstoss S.A., Plotkin L.I., Bellido T., Roodman G.D. (2017). Genetic deletion of Sost or pharmacological inhibition of sclerostin prevent multiple myeloma-induced bone disease without affecting tumor growth. Leukemia.

[17] Heath D.J., Chantry A.D., Buckle C.H., Coulton L., Shaughnessy J.D., Evans H.R., Snowden J.A., Stover D.R., Vanderkerken K., Croucher P.I. (2009). Inhibiting Dickkopf-1 (Dkk1) removes suppression of bone formation and prevents the development of osteolytic bone disease in multiple myeloma. J. Bone Miner. Res..

[18] Giuliani N., Morandi F., Tagliaferri S., Lazzaretti M., Donofrio G., Bonomini S., Sala R., Mangoni M., Rizzoli V. (2007). Production of Wnt inhibitors by myeloma cells: potential effects on canonical Wnt pathway in the bone microenvironment. Cancer Res..

[19] Lwin S.T., Edwards C.M., Silbermann R. (2016). Preclinical animal models of multiple myeloma. Bonekey Rep..

[20] Yaccoby S. (2010). Osteoblastogenesis and tumor growth in myeloma. Leuk. Lymphoma.

[21] Roodman G.D. (2009). Pathogenesis of myeloma bone disease. Leukemia.

[22] Yamaguchi A., Komori T., Suda T. (2000). Regulation of osteoblast differentiation mediated by bone morphogenetic proteins, hedgehogs, and Cbfa1. Endocr. Rev..

[23] Gartland A., Rumney R.M., Dillon J.P., Gallagher J.A. (2012). Isolation and culture of human osteoblasts. Methods Mol. Biol..

[24] Paton-Hough J., Tazzyman S., Evans H., Lath D., Down J.M., Green A.C., Snowden J.A., Chantry A.D., Lawson M.A. (2018 Oct 15). Preventing and repairing myeloma bone disease by combining conventional antiresorptive treatment with a bone anabolic agent in murine models. J. Bone Miner. Res..

[25] Hjertner O., Hjorth-Hansen H., Borset M., Seidel C., Waage A., Sundan A. (2001). Bone morphogenetic protein-4 inhibits proliferation and induces apoptosis of multiple myeloma cells. Blood.

[26] Derksen P.W., Tjin E., Meijer H.P., Klok M.D., MacGillavry H.D., van Oers M.H., Lokhorst H.M., Bloem A.C., Clevers H., Nusse R., van der Neut R., Spaargaren M., Pals S.T. (2004). Illegitimate WNT signaling promotes proliferation of multiple myeloma cells. Proc. Natl. Acad. Sci. U. S. A..

[27] Olsen O.E., Wader K.F., Misund K., Vatsveen T.K., Ro T.B., Mylin A.K., Turesson I., Stordal B.F., Moen S.H., Standal T., Waage A., Sundan A., Holien T. (2014). Bone morphogenetic protein-9 suppresses growth of myeloma cells by signaling through ALK2 but is inhibited by endoglin. Blood Cancer J..

[28] Laurikkala J., Kassai Y., Pakkasjarvi L., Thesleff I., Itoh N. (2003). Identification of a secreted BMP antagonist, ectodin, integrating BMP, FGF, and SHH signals from the tooth enamel knot. Dev. Biol..

[29] Clines K.L., Clines G.A. (2018). DKK1 and Kremen expression predicts the osteoblastic response to bone metastasis. Transl. Oncol..

[30] Mbalaviele G., Sheikh S., Stains J.P., Salazar V.S., Cheng S.L., Chen D., Civitelli R. (2005). Beta-catenin and BMP-2 synergize to promote osteoblast differentiation and new bone formation. J. Cell. Biochem..

[31] Silva A.C., Filipe M., Kuerner K.M., Steinbeisser H., Belo J.A. (2003). Endogenous Cerberus activity is required for anterior head specification in Xenopus. Development.

[32] Rawadi G., Vayssiere B., Dunn F., Baron R., Roman-Roman S. (2003). BMP-2 controls alkaline phosphatase expression and osteoblast mineralization by a Wnt autocrine loop. J. Bone Miner. Res..

[33] Ellies D.L., Viviano B., McCarthy J., Rey J.P., Itasaki N., Saunders S., Krumlauf R. (2006). Bone density ligand, Sclerostin, directly interacts with LRP5 but not LRP5G171V to modulate Wnt activity. J. Bone Miner. Res..

[34] Zhang R., Oyajobi B.O., Harris S.E., Chen D., Tsao C., Deng H.W., Zhao M. (2013). Wnt/beta-catenin signaling activates bone morphogenetic protein 2 expression in osteoblasts. Bone.

[35] Lintern K.B., Guidato S., Rowe A., Saldanha J.W., Itasaki N. (2009). Characterization of wise protein and its molecular mechanism to interact with both Wnt and BMP signals. J. Biol. Chem..

[36] Lyons K.M., Hogan B.L., Robertson E.J. (1995). Colocalization of BMP 7 and BMP 2 RNAs suggests that these factors cooperatively mediate tissue interactions during murine development. Mech. Dev..

