# Corrigendum to “Sostdc1: A soluble BMP and Wnt antagonist that is induced by the interaction between myeloma cells and osteoblast lineage cells” [Bone 122 (May 2019) 82–92]

**DOI:** 10.1016/j.bone.2019.04.020

**Published:** 2019-07

**Authors:** Z. Faraahi, M. Baud'huin, P.I. Croucher, C. Eaton, M.A. Lawson

**Affiliations:** a Institute for Cancer Sciences, University of Manchester, UK; b INSERM U957, EA 3822, Nantes, France; c Bone Biology Division, Garvan Institute of Medical Research, Sydney, Australia; d Department of Oncology and Metabolism, Medical School, University of Sheffield, UK

The authors regret that the original published acknowledgements were not complete. They should have read:

“The authors wish to thank in Bloodwise (formerly Leukaemia and Lymphoma Research) UK for their generous financial support for this work. ZF was also supported by an MRC UK studentship. We would also like to thank Dr Clive Buckle for his early gene expression studies on murine myelomas, which provided a preliminary identification of Sostdc1, as a gene of potential interest in myeloma-induced bone disease.”

The authors would like to apologise for any inconvenience caused.

