# Supplementary figures and images for: Sostdc1: A soluble BMP and Wnt antagonist that is induced by the interaction between myeloma cells and osteoblast lineage cells
Source: Bone. 2019 May;122:82–92. doi: 10.1016/j.bone.2019.02.012 (PMC6458996; doi:10.1016/j.bone.2019.02.012)

**A**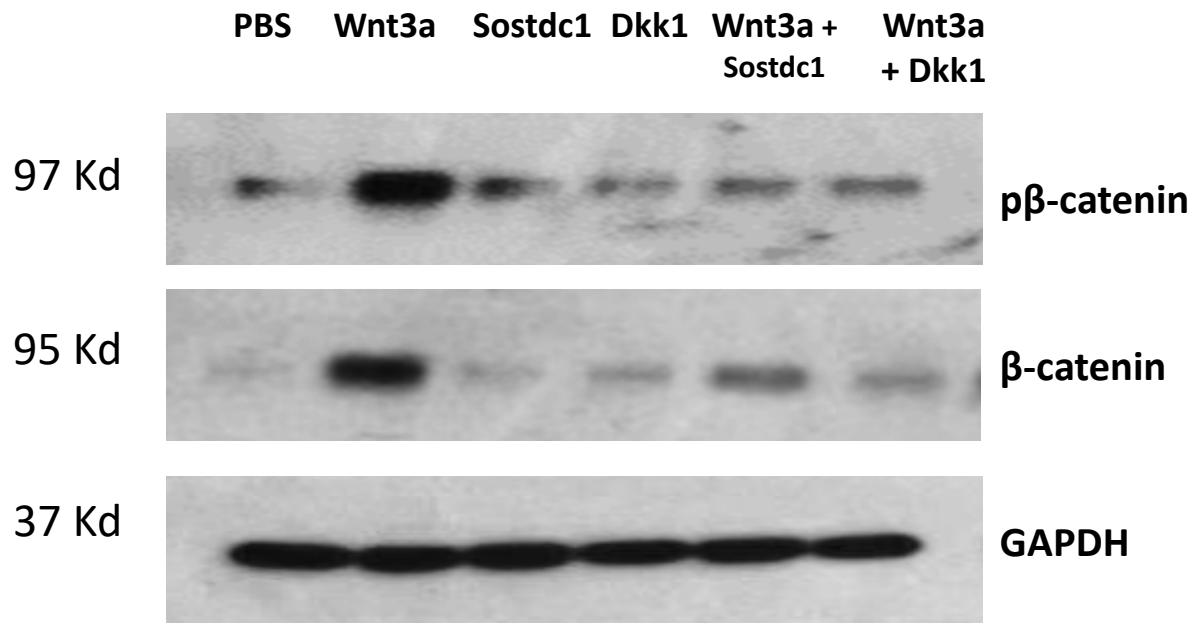**B**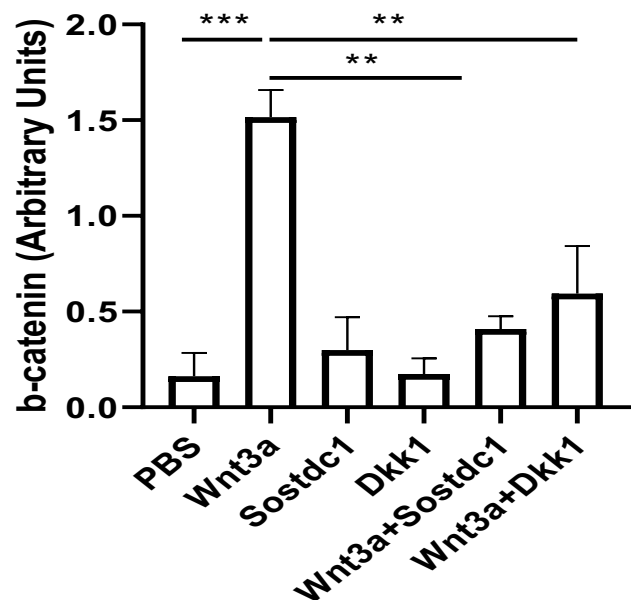**C**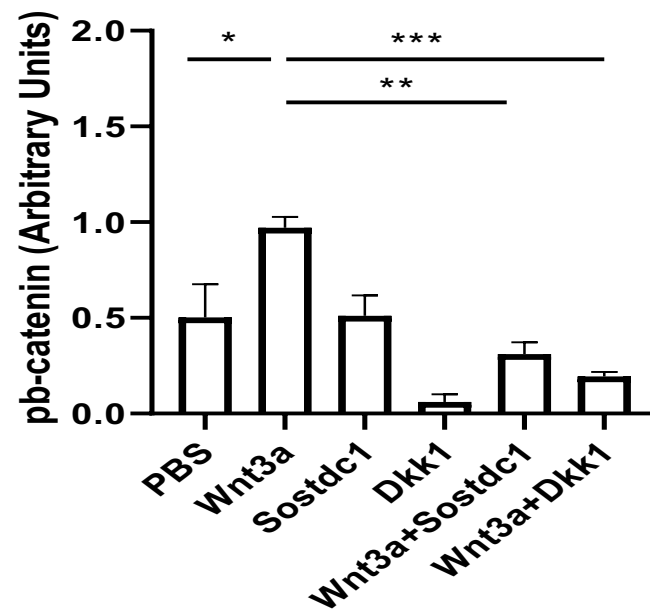

Supplement: Supplementary Fig. 1 — Sostdc1 suppressed Wnt3a-induced active and total β-catenin levels: Saos2 cells were treated with 50 ng/ml Wnt3a in the presence or absence of 250 ng/ml Sostdc1 or 100 ng/ml Dkk1 recombinant protein. Total and phosphorylated (p) β-catenin protein levels were assessed by western blotting (A). Multiple comparison analysis was used to assess the effect of Sostdc1 or Dkk1 on Wnt3a-induced active β-catenin (B) and pβ-catenin protein levels (C). Western blot image is representative of three independent experiments. One-way ANOVA, *P < 0.05, **P < 0.01 and ***P < 0.001. [file mmc1.pdf]
