# Supplementary material for: Sostdc1: A soluble BMP and Wnt antagonist that is induced by the interaction between myeloma cells and osteoblast lineage cells
Source: Bone. 2019 May;122:82–92. doi: 10.1016/j.bone.2019.02.012 (PMC6458996; doi:10.1016/j.bone.2019.02.012)
Supplement: Supplementary file 2 — Supplementary material [file mmc2.docx]

**Method**

Saos2 (ATCC® HTB-85™ ) cells were seeded at 300,000 cells per well in a 6 well culture plates in DMEM media for 24 h. Cells were starved overnight in DMEM containing no FCS. Wells containing cells were treated for 20 minutes with either PBS, 50 ng/ml recombinant Wnt3a, 250 ng/ml, Sostdc1 or 100 ng/ml Dkk1. Cells were also treated with Wnt3a in combination with Sostdc1 or Dkk1 at the concentrations mentioned above. Treated cells were lysed and 10 µg cell extracts gel-resolved and transferred onto a PVDF membrane for 1 h at 70 volts in 4ᴼC. Membranes were probed for total β-catenin (5 µg/ml, anti-β-catenin rabbit polyclonal antibody, Abcam), phosphorylated (p) β-catenin (8 µg/ml, anti-pβ-catenin rabbit polyclonal antibody, Abcam) and GAPDH (2 µg/ml, anti-GAPDH mouse monoclonal antibody, Abcam). A chemiluminescent substrate (Super Signal West Dura, Thermo Fisher) was used for detection of specific proteins on X-ray films (Kodak). GAPDH was used as loading control. Band intensities were assessed on ImageJ software version 1.6 (http://rsb.info.nih.gov/ij/; National Institutes of Health, Bethesda, MD, USA).

**Results**

Saos2 cells were treated Wnt3a in the presence or absence of Sostdc1 or Dkk1. Total and active (phosphorylated) β-catenin protein levels were assessed using western blot analysis (A). A One-Way Anova Bonferroni’s multiple analysis was used to assess the effect of Sostdc1 and Dkk1 on Wnt3a-induced active β-catenin (B) and phosphorylated (p) β-catenin protein levels (C). The total β-catenin protein levels in Saos2 cells were increased 2 fold by treatment with Wnt3a (p=0.0002) and this effect was suppressed in these cells in the presence of Sostdc1 (p=0.001) or Dkk1 (p=0.005). In addition, here we show that the levels of phosphorylated β-catenin are increased in the presence of Wnt3a compared to untreated controls (p=0.02). This effect was decreased by the presence of Sostdc1 (p=0.001) or Dkk1 (p=0.003). These results suggest that the presence of Wnt activated β-catenin was suppressed in Saos2 cells by treatment with either Sostdc1 or Dkk1.

**Supplementary Figure 1** – Sostdc1 supressed Wnt3a-induced active and total β-catenin levels: Saos2 cells were treated with 50 ng/ml Wnt3a in the presence or absence of 250 ng/ml Sostdc1 or 100 ng/ml Dkk1 recombinant protein. Total and phosphorylated (p) β-catenin protein levels were assessed by western blotting (A). Multiple comparison analysis was used to assess the effect of Sostdc1 or Dkk1 on Wnt3a-induced active β-catenin (B) and pβ-catenin protein levels (C). Western blot image is representative of three independent experiments. One-way Anova, *P<0.05, **P<0.01 and ***P<0.001.
